# Genetic Susceptibility in Sinusoidal Obstruction Syndrome/Veno-Occlusive Disease: A Case–Control Study

**DOI:** 10.3390/ijms26146712

**Published:** 2025-07-12

**Authors:** Ioulia Mavrikou, Marta Castelli, Tasoula Touloumenidou, Zoi Bousiou, Evangelia-Evdoxia Koravou, Anna Vardi, Apostolia Papalexandri, Christos Demosthenous, Maria Koutra, Paschalis Evangelidis, Alkistis-Kyra Panteliadou, Ioannis Batsis, Dimitrios Chatzidimitriou, Emmanouil Nikolousis, Alessandro Rambaldi, Ioanna Sakellari, Eleni Gavriilaki

**Affiliations:** 1 Hematology & BMT Unit, General Hospital “George Papanikolaou”, 57010 Thessaloniki, Greece; iouliamavrikou@gmail.com (I.M.); tasoula.touloumenidou@gmail.com (T.T.); boussiou_z@hotmail.com (Z.B.); evakikor@gmail.com (E.-E.K.); anna_vardi@yahoo.com (A.V.); lila.papalexandri@gmail.com (A.P.); christosde@msn.com (C.D.); tmdmgr@gmail.com (M.K.); kirapanteliadou@gmail.com (A.-K.P.); iobats@yahoo.gr (I.B.); ioannamarilena@gmail.com (I.S.); 2 Hematology and Bone Marrow Transplant Unit, Ospedale Papa Giovanni XXIII, 24127 Bergamo, Italyalessandro.rambaldi@unimi.it (A.R.); 3 Second Propedeutic Department of Internal Medicine, Hippocration Hospital, Aristotle University of Thessaloniki, 54642 Thessaloniki, Greece; pascevan@auth.gr; 4 Microbiology Department, Aristotle University of Thessaloniki, 54124 Thessaloniki, Greece; dihi@auth.gr; 5 Department of Haematology and BMT, American Hospital Dubai, Dubai, United Arab Emirates; info@drnikolousis.gr

**Keywords:** allogeneic hematopoietic stem cell transplantation, complement, defibrotide, genetic susceptibility, next-generation sequencing, sinusoidal obstruction syndrome (SOS), veno-occlusive disease (VOD)

## Abstract

Sinusoidal Obstruction Syndrome/Veno-Occlusive Disease (SOS/VOD) is a severe complication of hematopoietic cell transplantation (HCT). Furthermore, emerging evidence suggests the potential role of complement activation and endothelial injury in SOS/VOD pathogenesis. In this study, we aimed to identify potential distinct pathogenic genetic variants between SOS/VOD and other endothelial injury syndromes following HCT, such as transplant-associated thrombotic microangiopathy (TA-TMA). For this aim, genomic DNA from 30 SOS/VOD patients and 30 controls with TA-TMA was analyzed. Using Next-Generation Sequencing (NGS), variants in complement-related genes (CFH, CFI, CFB, CFD, C3, CD55, C5, CD46, and thrombomodulin/THBD) and ADAMTS13 were examined. Out of 426 detected variants, 20 were classified as pathogenic. In SOS/VOD patients, variants were identified in ADAMTS13 (4), CFH (3), C3 (2), and CFB (1) genes. One of the variants has been recognized as the strongest genetic predictor of ADAMTS13 activity. Controls exhibited more variants in complement-related genes, particularly CFH, CFI, and C3. The genetic differences between SOS/VOD and TA-TMA highlight different pathogenic mechanisms, offering the potential for targeted risk assessment and therapy in HCT recipients.

## 1. Introduction

Sinusoidal Obstruction Syndrome/Hepatic Veno-Occlusive Disease (SOS/VOD) is a severe complication that can arise from post-allogeneic hematopoietic cell transplantation (allo-HCT), alongside with other endothelial injury syndromes, such as transplant-associated thrombotic microangiopathy (TA-TMA) and graft-versus-host disease (GVHD) [1,2]. Although advancements in allo-HCT have resulted in a reduction in the syndrome’s incidence [3,4], several factors such as the use of calicheamicin-related antibodies, gemtuzumab, and inotuzumab ozogamicin have reignited clinical interest due to their association with increased SOS/VOD risk [5,6]. Until today, defibrotide administration has been suggested for the management of patients with moderate to severe SOS/VOD. Nevertheless, the exact mechanism of defibrotide action remains unclear [7,8], while given the increased mortality and morbidity of the syndrome, there is an unmet need for effective prophylactic interventions beyond ursodeoxycholic acid [9].

SOS/VOD pathophysiology is associated with endothelial injury, while damage to sinusoidal endothelial cells and hepatocytes leads to progressive venous obstruction [2]. The clinical features of SOS/VOD share common characteristics with a syndrome observed during pregnancy, HELLP syndrome (Hemolysis, Elevated Liver enzymes, and Low Platelets). Previous research by our group has identified similarities between SOS/VOD pathogenesis and other clinical entities, such as HELLP syndrome: in both syndromes, the increased activation of the complement system has been identified [10,11,12,13,14]. Additionally, studies have indicated that complement dysregulation plays a significant role in related conditions such as TA-TMA [15,16,17,18,19,20], and may also contribute to SOS/VOD [21,22,23]. Our group, along with others, has shown that alterations in coagulation and fibrinolysis are predictive of SOS/VOD development [24]. Different mutations and genetic alterations in complement-related genes may lead to distinct phenotypes with similar characteristics, as observed in other related endothelial injury syndromes [25,26]. Beyond complement activation, endothelial injury, along with the development of a procoagulant state, might have a substantial role in SOS/VOD pathogenesis, as shown in both experimental animal models and translational studies in allo-HCT recipients. In a recently published study investigating biomarkers for this syndrome, a SOS-VOD model was induced in female CD1 mice with the use of monocrotaline, and liver damage was identified 12 h post administration [27]. Histopathological analysis revealed platelet aggregation and the extravascular presence of von Willebrand factor (VWF) and ADAMTS13 (a disintegrin and metalloproteinase with a thrombospondin type 1 motif, member 13), indicating their association with SOS/VOD development, suggesting that the ADAMTS13 activity/concentration ratio can serve as an early biomarker for guiding therapeutic interventions. The activity of ADAMTS13, a well-known enzyme in the pathophysiology of TMAs, has been reported as decreased in patients with SOS/VOD [23]. Correspondingly, a mass spectrometry-based proteomics analysis identified potential biomarkers for SOS/VOD by comparing plasma samples from allo-HCT recipients with and without the condition [28]. A panel of five key biomarkers—the suppression of tumorigenicity-2 (ST2), angiopoietin2 (ANG2), L-Ficolin, hyaluronic acid (HA), and vascular cell adhesion molecule-1 (VCAM-1)—showed strong diagnostic potential. Notably, L-Ficolin, HA, and VCAM-1 were also effective in identifying patients at risk of developing SOS/VOD as early as the day of HCT. Another study evaluated 23 circulating plasma biomarkers after myeloablative allo-HCT to predict SOS/VOD risk before the emergence of clinical symptoms [29]. In a cohort of 33 cases matched with 107 controls, elevated levels of HA, ST2, ANG-2, L-ficolin, VCAM-1, intercellular adhesion molecule 1 (ICAM-1), TIMP metallopeptidase inhibitor 1 (TIMP-1), and thrombomodulin (TM) were linked to SOS/VOD risk. A similar study in a large pediatric cohort of 103 patients found that low L-Ficolin levels predicted SOS/VOD, while elevated ST2 and HA served as early prognostic biomarkers [30]. Moreover, a biomarker-driven fast-and-frugal decision tree (FFT) model has recently been developed to optimize preemptive defibrotide therapy for SOS/VOD in pediatric allo-HCT patients [31]. Using L-ficolin, HA, and stimulation 2 as biomarkers, the model effectively stratified patients by risk. When exploring potential biomarkers for SOS/VOD, patients with SOS/VOD had been found to be characterized by significantly higher baseline Endothelial Activation and Stress Index (EASIX), Fibrosis-4 (FIB-4), Aspartate Aminotransferase to Platelet Ratio Index (APRI), Albumin-Bilirubin (ALBI) grade, Model for End-Stage Liver Disease (MELD), and MELD-Na scores compared to non-SOS/VOD cases [32]. Thus, despite the several published studies in the field showing promising functional data regarding endothelial injury in SOS/VOD, evidence concerning the genetic susceptibility to this syndrome is scarce.

Interestingly, the concurrent development of TA-TMA and SOS/VOD has been associated with increased risk of progression to multi-organ dysfunction (MOD), indicating a potential biological interaction between the two conditions [33]. Consistent with these data, beyond TA-TMA, increased complement activation has been described in a patient with SOS/VOD who was effectively treated with a C1 complement inhibitor (C1-INH-C) [34]. Regarding genetic studies, Bucalossi et al. identified two CFH (complement factor H) variants in three patients with SOS/VOD. In addition to CFH and CFI (complement factor I), no other complement-related genes were studied in their cohort [35]. However, data regarding the distinct genetic susceptibility between SOS/VOD and TA-TMA are lacking.

Given the high mortality and morbidity that SOS/VOD patients experience, the necessity for the early recognition of the syndrome, and the lack of studies examining the genetic background of this syndrome, more studies investigating genetic susceptibility to SOS/VOD are crucial. Based on the above data, we aimed to identify genetic factors contributing to SOS/VOD development, recognizing the possible common or distinct pathways that this syndrome might share with TA-TMA. Thus, in this study, the genetic factors favoring SOS/VOD development were explored by comparing rare genetic variants in patients with SOS/VOD to those with TA-TMA to uncover the genetic susceptibility linked to complement dysregulation, and to provide valuable insights into the disease’s molecular pathogenetic mechanisms. In the era of precision medicine, the identification of these variants would enhance diagnostic precision, enable personalized prophylactic strategies, and guide early therapeutic interventions for high-risk patients undergoing allo-HCT.

## 2. Results

### 2.1. Study Population

We studied 30 patients with SOS/VOD and 30 patients with TA-TMA. The median age diagnosis of SOS/VOD patients was 45 (16–62) years and of TA-TMA controls 39 (19–57) (*p* = 0.191). The pre-transplant characteristics were similar between the groups, except for treatment with gemtuzumab or inotuzumab before allo-HCT, which was recorded in four patients with SOS/VOD, compared to one patient with TA-TMA (*p* = 0.03), and the use of concomitant calcineurin or the mechanistic target of rapamycin kinase (mTOR) inhibitors, which was more prevalent in TA-TMA patients (*p* < 0.001). An overview of the clinical characteristics of the studied population is presented in Table 1. Regarding the characteristics of SOS/VOD patients, the median time to SOS/VOD development was 22 (2–200) days post-allo-HCT. None of the SOS/VOD patients were diagnosed with concurrent TA-TMA, and none of the TA-TMA patients with SOS/VOD.

### 2.2. Genetic Analysis

A total of 426 variants were recorded after removing duplicates, from which 20 pathogenic variants were identified. In relation to SOS/VOD, pathological variants in the *ADAMTS13*, *CFH*, *C3*, and *CFB* genes were identified, as presented in Table 2.

More specifically, four variants were identified in the *ADAMTS13* gene (rs28647808 in five patients, rs28503257 in one patient, rs36090624 in one patient, and rs41314453 in two patients), three in *CFH* (rs35343172 in one patient, rs35274867 in one patient, and rs3753396 in six patients), two in *C3* (rs117793540 and rs149850773 in one patient each), and one variant in *CFB* (rs12614 in ten patients).

In patients with TA-TMA, a higher frequency of variants was observed in complement-related genes, including *CFH* genes (rs148403790 in two patients, rs149474608 in one patient, rs145975787 in one patient, rs513699 in three patients, rs61822181 in one patient, and rs460897 in two patients), *CFI* (rs7437875, rs114013791, rs41278047, and rs146444258 in one patient each), and *C3* (transcript ENST00000245907 in one patient), which are involved in complement-related disorders. The pathogenic variants detected in patients with TA-TMA are presented in Table 3.

To further understand the clinical significance of the detected variants in SOS/VOD patients, four bioinformatics tools were used. Variants predicted to be deleterious by four tools are presented in Table 4, those predicted by three tools are shown in Table 5, and those predicted by two tools are displayed in Table 6.

## 3. Discussion

To our knowledge, this is one of the first studies published examining genetic susceptibility to SOS/VOD in allo-HCT recipients, along with the potentially distinct genetic background of this syndrome with TA-TMA. Novel insights into the genetic susceptibility of SOS/VOD are presented, emphasizing the role of complement activation and coagulation pathways. We comprehensively analyzed key complement-related and coagulation-associated genes, identifying distinct pathogenic variants in SOS/VOD patients. Notably, a significant variant in ADAMTS13 emerged as the strongest genetic predictor of its activity, reinforcing its potential role in SOS/VOD pathogenesis. Our findings reveal fundamental genetic differences between SOS/VOD and TA-TMA, suggesting distinct underlying mechanisms. These results not only enhance our understanding of SOS/VOD etiology, but also pave the way for more precise risk stratification and targeted therapeutic strategies in HCT patients.

Historically, the diagnosis of SOS/VOD was made according to the Baltimore [36] or Seattle [37] criteria, after excluding other syndromes. The main difference between the two traditional criteria is the mandatory hyperbilirubinemia in the Baltimore criteria, which implies a longer waiting time for its development or inherently more aggressive forms. The clinical scenario can vary and change dynamically, especially in the pediatric population [38]. For these reasons, the European Society for Blood and Marrow Transplantation (EBMT) proposed novel, distinct diagnostic criteria and a scale for grading the severity of suspected SOS/VOD [38,39]. The EBMT criteria have been associated with severity grading scales related to dynamic changes, primarily the progression of hepatic and renal function tests, and were implemented in our study population. The speed of these changes is considered a warning sign that belongs to a higher severity grading scale (for suspected SOS/VOD), and thus supports the early initiation of treatment, potentially improving clinical outcomes. This grading system may also be used in cases of suspected SOS/VOD before patients meet diagnostic criteria, particularly before day 21 [39].

Jodele et al. were the first to propose that endothelial dysfunction syndromes arise following multiple stimuli in genetically predisposed pediatric patients with TA-TMA [15,40]. The initial data showed increased terminal complement pathway activation via a crude marker of sC5b-9 [40]. Further studies have also confirmed complement activation on the cell surface through functional methods [16]. Additionally, genetic data have highlighted genetic predisposition through rare mutations in complement-related genes [15]. Our group has confirmed these findings in adult patients [41], providing additional evidence of the vicious cycle of endothelial dysfunction, hypercoagulability, neutrophil activation, and complement activation [17]. A more recent RNA sequencing study in pediatric TA-TMA demonstrated the activation of multiple complement pathways and the interaction between complements and interferons, prolonging endothelial damage in TA-TMA [42]. Given the potential common pathophysiology of TA-TMA and SOS/VOD, in this case–control study, the role of genetic variants in complement-related genes in these clinical entities was investigated.

Our understanding of TA-TMA pathophysiology has revolutionized the management of these patients. Based on their success in patients with other complement-related TMAs and overt complement activation in TA-TMA [43,44], complement inhibitors have also shown safety and efficacy in this complication following allo-HCT. The first inhibitor of the complement terminal pathway, eculizumab (C5 inhibitor), has long been used in TA-TMA [45,46,47,48]. Real-world clinical data suggest the early initiation of therapy in patients with complement activation, assessed via sC5b-9 levels, as well as the monitoring of treatment and dose adjustments [49]. Additionally, a new lectin pathway inhibitor targeting MASP-2 (mannan-binding lectin-associated serine protease-2), narsoplimab, is being investigated for Food and Drug Administration (FDA) approval for the treatment of TA-TMA [50].

Although not all variants recognized in our study have been fully characterized, they have been previously described in relation to complement, thrombotic, and autoimmune disorders. Of particular interest is the rs41314453 variant, which has also been identified as the strongest genetic prognostic marker of ADAMTS13 activity. To elaborate further, concerning ADAMTS13 variants and more specifically rs28647808, a study involving 1163 patients with type 2 diabetes mellitus investigated whether this single nucleotide polymorphism (SNP) is associated with increased renal or cardiovascular complications, and its role in treatment responses to therapies aimed at mitigating these risks [51]. Patients were randomized to either ACE inhibitor (ACEi) therapy or a placebo, and renal and cardiovascular outcomes were assessed. The findings indicated that untreated carriers of the 618Ala allele (rs28647808[G]) had an approximately 50% higher risk of renal complications, such as progression to microalbuminuria, compared to Pro/Pro homozygotes. However, 618Ala carriers responded twice as effectively to ACEi therapy, with a reduced progression to renal endpoints (~3% vs. ~6% for Pro/Pro patients treated with ACE inhibitors). Moreover, regarding rs28503257, a study aimed to investigate the relationships between ADAMTS13 gene polymorphisms and atrial fibrillation (AF)—induced by arterial hypertension—enrolling a total of 200 hypertensive patients without AF (hypertension group) and 200 hypertensive patients with AF (AF group) [52]. The associations of polymorphisms of the rs28503257 loci in the ADAMTS13 gene with the clinical indexes of patients were investigated. The clinical indicators of patients were compared, and it was discovered that the ADAMTS13 rs28503257 polymorphism was related to brain natriuretic peptide (BNP) and D-dimer levels. The distribution of genotypes for ADAMTS13 rs28503257 (*p* = 0.047) and rs34054981 (*p* = 0.013) differed between the atrial fibrillation (AF) group and the hypertension group. The AF group had a lower frequency of the GA genotype of ADAMTS13 rs28503257 and a higher frequency of the CT genotype of ADAMTS13 rs34054981 compared to the hypertension group.

Regarding rs41314453 ADAMTS13, a genome-wide association study (GWAS) of ADAMTS13 activity has been performed, using imputed genotypes of common variants in a discovery sample of 3443 individuals and a replication sample of 2025 individuals [53]. In both the discovery and replication samples, rs41314453 was associated with ADAMTS13 activity and was identified as the main genetic determinant of ADAMTS13 activity. Furthermore, genetic predictors of ADAMTS-13 activity were derived from a GWAS, involving 5448 individuals of European ancestry, in order to evaluate the role of ADAMTS13 in ischemic heart disease [54]. The SNP rs41314453, which is functionally relevant to ADAMTS13 activity, was found to be inversely related to ischemic heart disease (IHD). The role of ADAMTS13 genetic variants in the prediction of the long-term cardiovascular outcomes of allo-HCT recipients should be further evaluated, given the increased cardiovascular disease burden that they experience [55,56,57].

Concerning rs35343172 CFH, in a study, multimodal imaging was used to identify phenotypic differences between carriers and noncarriers of rare CFH variants in age-related macular degeneration (AMD) patients [58]. The aim was to determine the association between rare genetic variants in CFH and phenotypic features in AMD patients. Phenotypic differences between carriers and noncarriers of rare variants in the CFH gene were identified, showing that carriers exhibited more severe disease. Selected AMD patients may also benefit from the use of complement inhibitors [59].

According to rs35274867 CFH, in a study of 30 Caucasian preeclamptic pregnant women, genetic sequencing of ADAMTS13 and complement regulatory genes was conducted, revealing that risk factor variants were found in the genes of ADAMTS13, C3, thrombomodulin, CFB, CFH, MBL2, and, finally, MASP2 [60]. Of five deleterious genetic variants that were identified in seven pregnant women suffering from preeclampsia, one was associated with the gene of CFH (rs35274867), linked with the occurrence of preeclampsia. In addition, the protective rare variant missense SNP rs35274867 was significantly associated with a decrease in the risk of advanced AMD [61].

Concerning rs3753396 CFH, the G allele of rs3753396 has been associated with the exudative form of AMD in a cohort of Chinese patients [62]. Furthermore, a case–control study of 130 unrelated native Northern Spanish individuals diagnosed with AMD was conducted to clarify the potential role of SNPs in the CFH gene in this clinical entity [63]. The haplotypes CGG (rs3753394, rs529825, and rs800292) and GCAG (rs203674, rs1061170, rs3753396, and rs1065489) were significantly associated with AMD, while the haplotypes CAA (rs3753394, rs529825, and rs800292) and TTAG (rs203674, rs1061170, rs3753396, and rs1065489) were found to be protective. Additionally, in a GWAS, which was followed by replication and meta-analysis, 2245 AMD patients and controls were included, concluding that rs3753396 SNP in CFH was associated with systemic complement activation levels [64].

Regarding rs117793540 C3, in a study of 81 adult Sickle Cell Disease (SCD) patients conducted by our group, 25 rare variants were detected, while rare variant rs117793540 was identified and characterized as pathogenic in the C3 gene [65]. Complement activation has been recognized in SCD patients and correlated with disease complications, such as transfusion-related hemolytic reactions [66,67]. Additionally, a study aimed to identify highly penetrant damaging mutations in genes associated with systemic lupus erythematosus (SLE)-like disease [68]. From the four main signaling pathways that were defined, the one that referred to immune complex clearance led to a mono-allelic variant in C3. Patients carrying this variant are predicted to experience a gain-of-function (GOF) (rs117793540) and the hyperactivation of C3 through the alternative C3-convertase pathway, disrupting the anaphylatoxin-mediated response to infection.

Regarding rs12614 CFB, a study was conducted to confirm the genetic influence of rs12614 on chronic hepatitis B (CHB) susceptibility and identify potential additional causal variants surrounding rs12614 in a Korean population [69]. In total, 10 genetic polymorphisms of CFB were chosen and analyzed in a study group consisting of 1716 individuals, comprising 955 CHB patients and 761 population controls. Rs12614 exhibited a notable genetic impact on the risk of chronic hepatitis B (CHB) within the Korean population.

There are several limitations that should be acknowledged in our study. Firstly, the control group was compromised by patients diagnosed with another endothelial injury syndrome (TA-TMA). Future research approaches should focus on genetic comparisons between not only patients with different endothelial injury syndromes, but also between healthy controls and patient donors. While we investigated genetic susceptibility, variations in genetic backgrounds across different populations may influence the reproducibility of our findings. Moreover, our sample size was relatively small, and larger studies in the field are needed to validate our results. Another interesting idea for future research approaches would be to additionally validate our findings in larger independent cohorts or public genetic databases. Finally, a limitation of our study is the lack of functional experiments and mechanistic data to further support the role of endothelial injury, coagulation, and complement system activation in SOS/VOD. These hypotheses should be further evaluated in future research.

## 4. Materials and Methods

### 4.1. Study Design and Patient Population

The inclusion criteria of the study were the following:

- Age ≥ 16 years old;
- Recipient of allo-HCT;
- Diagnosis of either SOS/VOD or TA-TMA;
- Availability of serum, plasma, and DNA samples;
- Informed patient consent.

Patients whose electronic medical records had missing data or did not provide informed consent were excluded from our study. Moderate, severe, or very severe SOS/VOD was diagnosed based on the revised EBMT criteria: bilirubin ≥ 2 mg/mL and two of the following criteria: painful hepatomegaly, weight gain, or ascites, within 21 days after allo-HCT [39]. Patients underwent allo-HCT at the collaborating transplant centers following the common guidelines and procedures recommended by the EBMT Secretariat [70]. The clinical data of the patients before and after allo-HCT were retrospectively recorded in a common form by all collaborating transplant centers. Serum, plasma, and DNA samples were retrospectively collected at the Molecular Biology Laboratory of the General Hospital of Thessaloniki “G. Papanikolaou” from patients who underwent allo-HCT during the last decade. The study was approved by the Scientific Committees of the respective centers and was conducted in accordance with the principles of the Declaration of Helsinki.

### 4.2. Genetic Analysis

Prior to allo-HCT, peripheral blood samples were collected and genomic DNA isolated. Genetic predisposition was analyzed using Next-Generation Sequencing (NGS) for complement-related genes (complement factor H/CFH, CFH-related, CFI, CFB, CFD, C3, CD55, C5, CD46, and thrombomodulin/THBD) and ADAMTS13. The investigation of complement-related genes and the ADATS13 gene was based on the speculation that complement activation and thrombosis might have an important role in SOS/VOD pathogenesis, similar to TA-TMA, given that both syndromes are characterized by endothelial injury and activation [17,41]. The primers and probes were designed in DesignStudio (Illumina) to cover the exons of the genes and an additional 15 bases within the intronic regions (98% coverage). For library preparation, 10 ng of genomic DNA was used (MiSeq, Illumina). The libraries were quantified using Qubit and sequenced on a MiSeq system in a 2 × 150 bp run (Illumina, San Diego, CA, USA). Both Ensembl and RefSeq resources were used for annotating the output files. The identified variants were evaluated using four bioinformatics tools. Those predicted to be deleterious by at least two tools (50%) were categorized as pathogenic. The clinical significance of the variants was also investigated using the current version of the complement database [25,71,72,73].

### 4.3. Bioinformatics Analysis

The complement database and ClinVar were used for the categorization of the variants. The Ensembl Variant Effect Predictor (VEP) tool was used to detect rare variants with a minor allele frequency (MAF) of less than 1% (0.01) in the European combined population of 1KG. Additionally, all identified genetic variants were compared with the 2018 version of the gene variant database, and those predicted to be deleterious by at least two tools (50%) were considered pathogenic. The following four bioinformatics tools were used to assess the clinical significance of the variants:

1. Sorting Intolerant From Tolerant (SIFT): SIFT predicts whether an amino acid substitution is deleterious, meaning it affects the protein function based on sequence homology and amino acids’ physical properties. Specifically, the “SIFT dbSNP” tool was used, which annotates and provides damaging/tolerated predictions for single nucleotide variants. A SIFT_SCORE ranging from 0 to 1 predicted that the amino acid substitution is deleterious if the score is ≤0.05, and tolerated if the score is >0.05;
2. Polymorphism Phenotyping v2 (PolyPhen-2): PolyPhen-2 is a software tool used to predict the possible impact of substitutions of amino acids on the structure and function of human proteins using precise physical and evolutionary comparative considerations [74]. Variants that are predicted to be deleterious are characterized by the tool as possibly damaging or probably damaging. Probably damaging is a more confident prediction;
3. Protein Variation Effect Analyzer (PROVEAN): PROVEAN (https://provean.jcvi.org/, accessed on September 2024) is a software tool predicting the possibility of the effect of an amino acid replacement or acid on the proteinaceous biological function. Protein variants with PROVEAN scoring equal or below a predefined threshold (we selected the default threshold −2.5) are predicted to have a “deleterious” effect [75];
4. Combined Annotation Dependent Depletion (CADD): CADD is a tool for scoring the deleteriousness of single nucleotide variants and insertion/deletion variants in the human genome [76]. A scaled C-score (PHRED-like) is calculated based on which variants are ranked relatively to all possible substitutions that can be performed for the human genome. Variants with a scaled C-score greater than or equal to 20 were selected, which indicates the 20% most deleterious substitutions.

### 4.4. Statistical Analysis

Sample size calculation was based on a previously published study of our group regarding the pre-transplant genetic susceptibility to TA-TMA in allo-HCT recipients [41]. The lack of relevant studies on SOS/VOD limited further estimations. According to our previous findings, a sample size of 60 patients would provide 80% power to detect a statistically significant difference at the 0.05 level. The descriptive analysis of the data was conducted using the SPSS 22.00 software package (Statistical Package for Social Sciences, version 22.00) for Windows. Results were expressed as frequencies and percentages for qualitative variables. The continuous variables’ normality tests used were: (a) the Kolmogorov–Smirnov test (samples with statistical significance *p* < 0.05 were considered non-normally distributed), (b) histogram and boxplot inspection (visual assessment), and (c) skewness and kurtosis indices (values between −1 and +1 were considered indicative of normal distribution). Non-normally distributed data were presented with the median and interquartile range (Q1–Q3).

The comparison between qualitative variables was performed using the chi-square test. Differences between means were calculated using Student’s *t*-test for two independent samples, following the Levene’s test for equality of variances between the two groups. If at least one variable was not normally distributed, the non-parametric Mann–Whitney test was used to compare the means between the two groups.

## 5. Conclusions

This study revealed pathogenic genetic variants in pre-transplantation blood samples from patients who developed SOS/VOD, highlighting the role of multiple pathways related to the endothelium, coagulation, and complement. The pathogenic genetic variants identified in patients with TA-TMA were not shared with those observed in patients with SOS/VOD, suggesting distinct mutation patterns between the groups. The correlation of these variants with phenotypic characteristics and further investigation into the effects of variant combinations are required. In the future, we plan to investigate the role of these genetic variants in the prediction of outcomes and overall survival in SOS/VOD patients. Moreover, whole genome sequencing analysis in SOS/VOD patients can be helpful for this aim [77].

## Figures and Tables

**Table 1:** An overview of the clinical characteristics of SOS/VOD patients and TA-TMA controls.

Characteristics	SOS/VOD (*n* = 30)	TA-TMA (*n* = 30)	*p*-Value
**Age at Diagnosis, Median (IQR), Years**	45 (16–62)	39 (19–57)	0.191
**Gender—Male, *n* (%)**	12 (40)	11 (36.7)	0.791
**Primary disease**			0.791
AML, *n* (%)	12 (40)	10 (33.3)	
ALL, *n* (%)	7 (23.4)	12 (40)	
MDS, *n* (%)	4 (13.3)	3 (10)	
MPN, *n* (%)	4 (13.3)	3 (10)	
NHL, *n* (%)	3 (10)	2 (6.7)	
**Treatment with gemtuzumab or inotuzumab prior to allo-HCT**	4 (13.2)	1 (3.3)	0.003
**Donor type**			0.960
HLA-matched sibling, *n* (%)	6 (20)	5 (16.7)	
Matched unrelated, *n* (%)	4 (13.4)	5 (16.7)	
Mismatched unrelated, *n* (%)	10 (33.3)	9 (30)	
Haploidentical, *n* (%)	10 (33.3)	11 (36.6)	
**Myeloablative conditioning, *n* (%)**	21 (70)	23 (76.7)	0.766
**Concomitant calcineurin or mTOR inhibitors, *n* (%)**	5 (16.7)	20 (66.7)	<0.001
**Grade II** **–IV acute/chronic GVHD, *n* (%)**	26 (86.7)/13 (43.3)	25 (83.4)/16 (53.3)	0.718/0.438

ALL = acute lymphoblastic leukemia; allo-HCT = allogenic hematopoietic cell transplantation, AML = acute myeloid leukemia; GVHD = graft-versus-host disease, HLA = human leukocyte antigen, IQR = interquartile range; mTOR = mechanistic target of rapamycin kinase, and SOS/VOD = Sinusoidal Obstruction Syndrome/hepatic Veno-Occlusive Disease.

**Table 2:** Variants detected in patients with SOS/VOD.

SOS/VOD	
**Gene ADAMTS13**	
rs28647808	5 patients
rs28503257	1 patient
rs36090624	1 patient
rs41314453	2 patients
**Gene CFH**	
rs35343172	1 patient
rs35274867	1 patient
rs3753396	6 patients
**Gene C3**	
rs117793540	1 patient
rs149850773	1 patient
**Gene CFB**	
rs12614	10 patients

ADAMTS13 = a disintegrin and metalloproteinase with a thrombospondin type 1 motif, member 13; C3 = complement factor 3; CFB = complement factor B; CFH = complement factor H; and SOS/VOD = Sinusoidal Obstruction Syndrome/Veno-Occlusive Disease.

**Table 3:** Variants detected in patients with TA-TMA.

TA-TMA	
**Gene CFH**	
rs148403790	2 patients
rs149474608	1 patient
rs145975787	1 patient
rs513699	3 patients
rs61822181	1 patient
rs460897	2 patients
**Gene CFI**	
rs7437875	1 patient
rs114013791	1 patient
rs41278047	1 patient
rs146444258	1 patient
**Gene C3**	
ENST00000245907	1 patient

C3 = complement factor 3; CFH = complement factor H; CFI = complement factor I; and TA-TMA = transplant-associated thrombotic microangiopathy.

**Table 4:** Variants predicted to be deleterious by four tools.

*n*	Variant	Gene	SIFT_Prediction	Polyphen_Prediction	PROVEAN_Prediction	# Tools	# Patients
1	rs5030737	MBL2	DELETERIOUS	Probably damaging	Deleterious	4	6
2	rs1800450	MBL2	DELETERIOUS	Probably damaging	Deleterious	4	13
3	rs1800451	MBL2	DELETERIOUS	Probably damaging	Deleterious	4	2

MBL2 = Mannose-Binding Lectin 2.

**Table 5:** Variants predicted to be deleterious by three tools.

*n*	Variant	Gene	SIFT_Prediction	Polyphen_Prediction	PROVEAN_Prediction	# Tools	# Patients
4	rs28647808	ADAMTS13	DELETERIOUS	Probably damaging	Neutral	3	5
5	rs12614	C2,C2-AS1,CFB	DELETERIOUS	Probably damaging	Neutral	3	10
6	rs28503257	ADAMTS13	DELETERIOUS	Probably damaging	Neutral	3	1
7	rs35343172	CFH	DELETERIOUS	Possibly damaging	Deleterious	3	1
8	rs36090624	ADAMTS13	DELETERIOUS	Probably damaging	Neutral	3	1
9	rs117793540	C3	DELETERIOUS	No prediction	Deleterious	3	1
10	rs149850773	C3	DELETERIOUS	No prediction	Deleterious	3	1
11	rs12711521	MASP2	DELETERIOUS	No prediction	Deleterious	3	52

ADAMTS13 = a disintegrin and metalloproteinase with a thrombospondin type 1 motif, C3 = complement factor 3, CFB = complement Factor B, CFH = complement factor H, and MASP2 = mannan-binding lectin serine protease 2.

**Table 6:** Variants predicted to be deleterious by two tools.

*n*	Variant	Gene	SIFT_Prediction	Polyphen_Prediction	PROVEAN_Prediction	# Tools	# Patients
12	rs72550870	MASP2	DELETERIOUS	No prediction	Neutral	2	2
13	rs139962539	MASP2	DELETERIOUS	No prediction	Deleterious	2	1
14	rs41314453	ADAMTS13	TOLERATED	Possibly damaging	Neutral	2	1
15	rs35274867	CFH	TOLERATED	Possibly damaging	Deleterious	2	1
16	rs3753396	CFH	TOLERATED	No prediction	Deleterious	2	20

ADAMTS13 = a disintegrin and metalloproteinase with a thrombospondin type 1 motif, CFH = complement factor H, and MASP2 = mannan-binding lectin serine protease 2.

## Data Availability

The data are available upon request from the corresponding author.

## Abbreviations

The following abbreviations are used in this manuscript:

ABLBI	Albumin-Bilirubin
ACEi	ACE inhibitor
ADAMTS13	A Disintegrin and metalloproteinase with a thrombospondin type 1 motif, member 13
AF	Atrial Fibrillation
ALL	Acute lymphoblastic leukemia
AMD	Age-related macular degeneration
AML	Acute myeloid leukemia
ANG2	Angiopoietin 2
APRI	Aspartate Aminotransferase to Platelet Ratio Index
BNP	Brain Natriuretic Peptide
C1-INH-C	C1 complement inhibitor
CADD	Combined Annotation Dependent Depletion
CFH	Complement Factor H
CFI	Complement Factor I
CHB	Chronic Hepatitis B
EASIX	Endothelial activation and stress index
EBMT	European Society for Blood and Marrow Transplantation
FDA	Food and Drug Administration
FFT	Fast-and-Frugal decision tree
FIB-4	Fibrosis-4
gDCA	Generalized decision curve analysis
GOF	Gain-of-function
GVHD	Graft-Versus-Host Disease
GWAS	Genome wide association study
HA	Hyaluronic acid
HCT	Hematopoietic cell transplantation
HELLP	Hemolysis, Elevated Liver enzymes, and Low Platelets
ICAM-1	Intercellular adhesion molecule 1
IHD	Ischemic heart disease
MAF	Minor Allele Frequency
MASP-2	Mannan-binding lectin-associated serine protease-2
MELD	Model for End-Stage Liver Disease
MOD	Multi-organ dysfunction
mTOR	Mechanistic target of rapamycin kinase
NGS	Next-Generation Sequencing
PolyPhen-2	Polymorphism Phenotyping v2
PROVEAN	Protein Variation Effect Analyzer
sC5b-9	Soluble C5b-9
SCD	Sickle Cell Disease
SD	Standard Deviation
SIFT	Sorting Intolerant From Tolerant
SLE	Systemic Lupus Erythematosus
SNP	Single nucleotide polymorphism
SOS/VOD	Sinusoidal Obstruction Syndrome/Veno-Occlusive Disease
ST2	Suppression of Tumorigenicity-2
TA-TMA	Transplant-associated thrombotic microangiopathy
THBD	Thrombomodulin
TIMP-1	TIMP metallopeptidase inhibitor 1
TM	Thrombomodulin
VCAM-1	Vascular cell adhesion molecule 1
VEP	Variant Effect Predictor
VWF	Von Willebrand Factor
